# Chlorogenic acid: A potent molecule that protects cardiomyocytes from TNF‐α–induced injury via inhibiting NF‐κB and JNK signals

**DOI:** 10.1111/jcmm.14351

**Published:** 2019-04-29

**Authors:** Lei Tian, Cong‐Ping Su, Qing Wang, Fu‐Jian Wu, Rui Bai, Hui‐Min Zhang, Jin‐Ying Liu, Wen‐Jing Lu, Wei Wang, Feng Lan, Shu‐Zhen Guo

**Affiliations:** ^1^ School of Traditional Chinese Medicine Beijing University of Chinese Medicine Beijing China; ^2^ Beijing Laboratory for Cardiovascular Precision Medicine Anzhen Hospital, Capital Medical University Beijing China

**Keywords:** apoptosis, chlorogenic acid, hiPSC‐CMs, tumour necrosis factor‐α

## Abstract

The traditional Chinese herb *Lonicerae Japonicae Flos* has shown significant clinical benefits in the treatment of heart failure, but the mechanism remains unclear. As the main active ingredient found in the plasma after oral administration of *Lonicerae Japonicae Flos*, chlorogenic acid (CGA) has been reported to possess anti‐inflammatory, anti‐oxidant and anti‐apoptosis function. We firstly confirmed the cardioprotective effects of CGA in transverse aortic constriction (TAC)‐induced heart failure mouse model, through mitigating the TNF‐α–induced toxicity. We further used TNF‐α‐induced cardiac injury in human induced pluripotent stem cell‐derived cardiomyocytes (hiPSC‐CMs) to elucidate the underlying mechanisms. CGA pre‐treatment could reverse TNF‐α–induced cellular injuries, including improved cell viability, increased mitochondrial membrane potential and inhibited cardiomyocytes apoptosis. We then examined the NF‐κB/p65 and major mitogen‐activated protein kinases (MAPKs) signalling pathways involved in TNF‐α–induced apoptosis of hiPSC‐CMs. Importantly, CGA can directly inhibit NF‐κB signal by suppressing the phosphorylation of NF‐κB/p65. As for the MAPKs, CGA suppressed the activity of only c‐Jun N‐terminal kinase (JNK), but enhanced extracellular signal‐regulated kinase1/2 (ERK1/2) and had no effect on p38. In summary, our study revealed that CGA has profound cardioprotective effects through inhibiting the activation of NF‐κB and JNK pathway, providing a novel therapeutic alternative for prevention and treatment of heart failure.

## 1 INTRODUCTION

Heart failure (HF) has been defined as a multifactorial degenerative disease, characterized by the impaired ability to fill the left ventricle and/or pump blood to satisfy the demands of the body. It is considered as the final common stage of numerous acute and chronic diseases, such as myocardial infarction, myocarditis, hypertension and diabetes. Despite advances in pharmacological or non‐pharmacological therapies, heart failure continues to be a global pandemic, directly affecting at least 26 million people globally annually, and its prevalence is increasing.^1^ Therefore, it is vital to develop novel therapeutic alternatives for preventing and treating heart failure.

Tumour necrosis factor‐alpha (TNF‐α) is a typical pro‐inflammatory cytokine that has a pivotal role in the pathological processes of heart failure.^2^ In 1990, Levine et al demonstrated a close relationship between the circulating levels of TNF‐α and the degree of heart failure.^3^ Furthermore, multiple studies have revealed that elevated TNF‐α levels are independent mortality predictors for patients with heart failure.^4^, ^5^ However, TNF‐α mediated effects are not always detrimental in the heart. As a stress‐response protein, it has a cytoprotective effect on the heart during ischemic injury and infectious myocarditis.^6^, ^7^ Nevertheless, when expressed at sufficiently high concentrations and persistently elevated, TNF‐α induces progressive left ventricular remodelling and dysfunction, cardiomyopathy, cardiomyocyte hypertrophy and apoptosis.^8^, ^9^, ^10^ Several lines of evidence have demonstrated that heart failure is related to the increased expression of TNF‐α–induced myocyte apoptosis. Gordon et al suggested that TNF‐α promoted progressive LV dysfunction in dog models of heart failure, mediated in part by increasing cardiomyocyte apoptosis.^11^ Additionally, TNF‐α antagonism has cardioprotective effects in experimental rat models of heart failure and a cardiac‐restricted TNF‐α overexpression mouse model.^12^, ^13^ Therefore, therapeutic approaches that block TNF‐α signals might have prophylactic value against heart failure, mainly though inhibiting myocyte apoptosis.^14^

TNF‐α–induced myocyte apoptosis involves complicated mechanisms.^15^ At the molecular level, the effect of TNF‐α on cardiomyocytes relies on binding to the TNF receptor and subsequent intracellular signalling via the mitogen‐activated protein kinases (MAPKs) and nuclear factor‐κB(NF‐κB) pathways.^16^, ^17^ MAPKs, which represent a family of cellular enzymes, may regulate the process of TNF‐α–induced apoptosis. The three major types of MAPKs are p38MAPK, extracellular signal‐regulated kinase1/2 (ERK1/2) and c‐Jun N‐terminal kinase (JNK).^18^ Previous studies showed that JNK and p38MAPK were related to cardiomyocyte apoptosis. However, ERK1/2 kinase is partly involved in cell survival and protected myocardium from ischemic damage.^19^ An imbalance between survival signals regulated by ERK1/2 and death signals generated by p38MAPK and JNK result in cell apoptosis. In addition, NF‐κB signalling pathway is also involved in TNF‐α induce apoptosis. TNF‐induced TNFR1 signalling results in NF‐κB activation, which was shown to participate in heart failure and pathological remodelling.^20^ Studies suggest that the NF‐κB/p65‐regulated phosphorylation of myosin light chain kinase (MLCK) and myosin light chain (MLC) alleviated the TNF‐α–induced injury of intestinal epithelial cells.^21^

*Lonicerae Japonicae Flos,* as a traditional medicinal herb known as ‘Jin Yin Hua', is used to treat various diseases including acute fever, headache and acute dysentery.^22^, ^23^ In addition, previous studies showed that *Lonicerae Japonicae Flos,* which was used in traditional Chinese formula, had significant benefits in the treatment of heart failure in clinical as well as animal research.^24^, ^25^ The effects of *Lonicerae Japonicae Flos* may be associated with its major active ingredient chlorogenic acid (5‐caffeoylquinic acid, CGA). Earlier studies indicated that CGA has anti‐inflammatory,^26^ anti‐oxidant,^27^ anti‐apoptotic,^28^ analgesic,^29^ antihyperalgesic^30^ and antidiabetic effects.^31^ However, despite the multifunctionality of CGA, little is known about its effect on heart failure. An in vivo study reported CGA might be useful to treat inflammation and ameliorate colitis severity by inhibiting TNF‐α expression and apoptotic signalling pathways.^32^ However, whether a decrease in TNF‐α induced by CGA is protective against cell apoptosis during heart failure is unclear.

Therefore, we investigated the effect of CGA on cardiovascular disease in a mouse model of TAC induced heart failure. The results of in vivo experiments show that CGA has cardioprotective effects and inhibited the high expression of TNF‐α in a heart failure mouse model. We used human induced pluripotent stem cell‐derived cardiomyocytes (hiPSC‐CMs) to explore whether CGA might have cardioprotective effects against the TNF‐α–induced apoptosis of myocardial cells and elucidate the underlying mechanism(s). Taken together, our findings demonstrated that CGA effectively alleviated TNF‐α overexpression induced injury in a TAC heart failure mouse model and protected hiPSC‐CMs from TNF‐α–induced apoptosis. Moreover, NF‐κB/p65 and JNK signals participated in the inhibitory effects of CGA on cardiomyocyte apoptosis.

## 2 MATERIALS AND METHODS

All animal experiments were performed in accordance with the ‘National Institute of guiding principles of the care and use of experimental animals' by the China Physiological Society. This study was approved by the Animal Research Ethics Committee of the Beijing University of Chinese Medicine (BUCM‐4‐2018060445‐2049). Male C57BL/6N mice (SCXK(Jing)2016‐0006) were provided by the Beijing Vital River Laboratory Animal Technology Co. Ltd. (Beijing, China) and raised in clean conditions at a temperature of 22 ± 1°C with 55 ± 5% humidity and a 12 hours light/dark cycle. After 1 week of adaptation, 27 C57BL/6N mice were randomly divided into four groups: (a) control group(n = 6); (b) sham + double distilled water (DDW)group (n = 6); (c) TAC + DDW group(n = 6): the TAC‐induced mice heart failure model was performed as previously described,^33^the mice of sham group animals underwent the same procedure but without aortic ligation; and (d) TAC + CGA group(n = 9). CGA was dissolved in DDW and administered intragastrically (110 mg/kg/d) in the TAC + CGA group for 28 days. In the sham + DDW and TAC + DDW groups, DDW was administered intragastrically every day. All mice had free access to tap water and food.

### 2.1 Echocardiographic evaluation of left ventricular function

Echocardiography was performed four weeks after the TAC operation using a Vevo 2100 ultrasound (Visualsonics, Toronto, ON, Canada). The centre‐frequency of the corresponding probe (MS‐400) was 30 MHz. Mouse chest hair was shaved and they were anaesthetized with isoflurane. Then the mice were put in a supine position. Two dimensional views of the left parasternal short axis and left ventricle in the long axis were assessed. In these views, 10 cardiac cycles were noted at every measured point. The bisecting, fractional shortening (FS) and ejection fraction (EF) were calculated though left ventricle (LV) and motion (m)‐mode measurements.

### 2.2 Histopathological assessment

The heart tissues of mice were fixed by 4% paraformaldehyde and dehydrated with different grades of ethanol. Then the heart tissues were embedded in paraffin and cut into 3‐μm sections. Tissue sections were deparaffinized by xylene, rehydrated via different grades of ethanol and stained with haematoxylin and eosin. Then digital images were observed under a microscope (Leica Biosystems Richmond, Inc).

### 2.3 Immunohistochemical staining for TNF‐α

Paraffin‐embedded cardiac tissue sections from different groups were deparaffinized by xylene and then rehydrated in different grades of ethanol. Then 3% H_2_O_2_ was added to the deparaffinized cardiac tissue sections for 20 minutes to reduce endogenous peroxidase activity. The sections were heated in a microwave in retrieval solution for 15 minutes to retrieve antigens. The slides were subsequently incubated in 10% goat serum for 2 hours at room temperature to block non‐specific binding. Then the slides were incubated with TNF‐α primary antibody (Abcam, ab6671) at 4°C overnight. The next day, the slides were incubated with secondary antibody (Gene Tex,GK500705) for 30 minutes at room temperature. Finally, they were visualized with 3,3′‐diaminobenzidine tetrahydrochloride (DAB) staining. Three slices from each group were randomly selected and semi‐quantitative image analysis using ImageJ software (National Institutes of Health, USA).

### 2.4 Culture and treatment of myocardial cells from urine human induced pluripotent stem cells

Urinary epithelial cell‐derived hiPSCs (Cellapy, Beijing, China) were cultured in PSCeasy hESCs/iPSCs medium (CA1001500; Cellapy). The cells were passaged every 3‐4 days at 80% confluence by ethylenediaminetetraacetic acid (EDTA) (HyClone) at 37°C and 5% CO_2_. The hiPSCs were assessed for pluripotency (Figure S1A).

Cardiac differentiation of hiPSCs was performed as previously reported^34^ with some modifications. A CardioEasy cardiac differentiation kit (CA2004500; Cellapy) was used for the differentiation of hiPSCs to cardiomyocytes. Briefly, when hiPSC reached ~ 80% confluency, media was changed for Induction Medium I. After 48 hours, hiPSCs were cultured in Induction Medium II for 48 hours and then in Induction Medium III, which was changed every other day. At days 7‐9, the cells began to contract. Then, hiPSC‐CMs were glucose starved for 3 days by CardioEasy purification (CA2005100; Cellapy). hiPSC‐CMs at days 28‐40 after cardiac differentiation were utilized for this study. hiPSC‐derived cardiomyocytes were identified by immunofluorescence staining (Figure S1B).

hiPSC‐CMs were dissociated by CardioEasy CM dissociation enzyme set (CA2006100; Cellapy; Beijing; China). Briefly, 1 mL of collagen‐based enzyme I was added to each 6‐well plate for 30 minutes at 37°C, and then 1 mL of trypsin‐based enzyme 2 was added for another 10‐20 minutes. Digested hiPSC‐CMs were seeded onto 6‐well plates by 5% KnockOut™ Serum Replacement (KSR) (Gibco Knock Out™, SR, USA).

For all trials in our study, hiPSC‐CMs were seeded at 80%‐90% density according to our experimental protocol. hiPSC‐CMs were randomly divided into four experimental groups: (a) control group: cardiomyocytes continuously cultured by 5% KSR; (b) TNF‐α group: cardiomyocytes treated with 40 ng/mL TNF‐α (PeproTech, Recombinant Human TNF‐α, 300‐01A) for 24 hours; (c) 0.1 μmol/L CGA treatment group: cardiomyocytes pre‐incubated with 0.1 μmol/L CGA (CHENGDU PUSH BIO‐TEC, China, PS0131‐0025) for 12 hours and then co‐incubated with CGA and TNF‐α for 24 hours; and (d) 1 μmol/L CGA: cardiomyocytes pre‐incubated with 1 µmol/L CGA for 12 hours and then co‐incubated with CGA and TNF‐α for 24 hours. All drugs were dissolved in pre‐warmed 5% KSR and then were added directly to hiPSC‐CMs. In the control groups, equivalent volumes of culture medium were added.

### 2.5 Immunostaining

Cells were grown on coverslips and then fixed with 4% paraformaldehyde for 30 minutes. Cells were blocked with 5% goat serum and 5% bovine serum albumin (BSA) at room temperature, and then incubated with primary antibodies: rabbit polyclonal anti‐OCT4 (1:100; Santa Cruz Biotechnology; sc‐9081), mouse monoclonal anti‐TRA‐1‐81 (1:100; Santa Cruz Biotechnology; sc‐21706), mouse monoclonal anticardiac troponin T (cTnT) (1:100; Abcam; Ab8295) and rabbit polyclonal anti‐α‐actinin (1:100; Abcam; Ab137346) at 4°C overnight. After washing with PBS, the slices were incubated with secondary antibodies: chicken antirabbit IgG Alexa Fluor 594 (1:200; Invitrogen; A21442), chicken antimouse IgG Alexa Fluor 488 (1:200; Invitrogen; A21200), goat antirabbit IgG Alexa Fluor 488 (1:200; Invitrogen; A32731) and goat antimouse IgG Alexa Fluor 594 (1:200; Invitrogen; A21145) at room temperature for 1 hour. Then the slices were rinsed in PBS three times and mounted with DNA‐specific 4′,6‐diamidino‐2‐phenylindole (DAPI; H1200; Vector Lab). Fluorescence was evaluated by a confocal laser scanning microscope (Leica, Wetzlar, Germany).

### 2.6 Cell survival assay

Cell viability was examined by a Cell Counting Kit‐8 (CCK‐8) (Dojindo, Kumamoto, Japan) to detect the cellular dehydrogenase activity of living cells. Briefly, different groups of hiPSC‐CMs in 96‐well gelatin‐coated plates received 10 μL CCK‐8 solution. Then the hiPSC‐CMs were incubated for 1‐4 hours at 37°C. An automatic microplate reader (Bio‐Tek Synergn4, Winooski, VT, USA) was used to evaluate the absorbance of each sample at 450 nm.

### 2.7 Flow cytometry assessment of apoptosis

The proportions of apoptotic hiPSC‐CMs in different treatment groups, including control group, TNF‐α group, CGA treatment group, and QNZ (NF‐κB Inhibition, Selleck, S4902) (QNZ concentration in hiPSC‐CMs was detected by CCK‐8 (Figure S1C)), SB203580 (P38 Inhibition, Selleck, S1076) (SB203580 concentration of hiPSC‐CMs was detected by CCK‐8 (Figure S1D)), SP600125 (JNK Inhibition, Selleck, S1460) (SP600125 concentration of hiPSC‐CMs was detected by CCK‐8 (Figure S1E)) and LY3214996 (ERK1/2 Inhibition, Selleck, S8534) (LY3214996 concentration of hiPSC‐CMs was detected by CCK‐8 (Figure S1F)) were evaluated by annexin V‐FITC and PI double‐staining assay kits (BD Pharmingen™ FITC Annexin V Apoptosis Detection Kit I, 556547) and flow cytometry as the per the manufacturer's instructions. Briefly, hiPSC‐CMs were cultured on 12‐well collagen‐coated plates. After the different treatments, hiPSC‐CMs from each group were harvested and then washed twice with cold PBS. Next, hiPSC‐CMs at a concentration of 1×10^5^ cells/mL were resuspended in 1 × Binding Buffer and then incubated with 5 μL FITC Annexin V and 5 μL PI for 15 minutes at RT (25°C) in the dark. The hiPSC‐CMs were incubated with 400 μL of 1 × Binding Buffer and analysed by flow cytometry (BD:LSRFortessa).

### 2.8 Evaluation of mitochondrial transmembrane potential

Changes in the mitochondrial membrane potential of different groups of hiPSC‐CMs were evaluated by JC‐1 staining (Solarbio^®^, M8650). hiPSC‐CMs were cultured on 96‐well collagen‐coated plates. Different groups of hiPSC‐CMs were washed with cold PBS and incubated with 100 μL JC‐1 work solution for 30 minutes at 37°C in the dark. The image was then scanned using a high‐content analyzer (Molecular Devices, USA), using the MetaXpress high‐content analysis system (ACEA Biosciences, USA) to set the cell parameters and automatically calculate the average fluorescence intensity of the cells.

### 2.9 Western blotting

After pre‐treatment with 0.1 μmol/L as well as 1 μmol/L CGA for 12 hours, followed by treatment with 40 ng/mL TNF‐α for 24 hours, the nuclear proteins and all cell lysates of hiPSC‐CMs were extracted using cell protein extraction kits. hiPSC‐CMs were treated with a protease and phosphatase inhibitor cocktail, and then a Bicinchoninic acid disodium (BCA) protein assay was performed to evaluate the protein concentrations (Thermo Scientific™ Pierce™ BCA Protein Assay Kit, 23227) according to the manufacturer's instructions. All samples were subsequently adjusted to the same volume by 2 × 4% sodium dodecyl sulfate (SDS) sample buffer and boiled for 5 minutes. Then equal amounts of protein were resolved by electrophoresis on 10% SDS‐PAGE. The fractionated proteins of all samples were transferred to a polyvinylidene fluoride (PVDF) (MerckMillipore NO:IPVH00010) membrane by electrophoresis for 90 minutes at 300 mA. The membranes were blocked with 5% non‐fat milk powder for 1 hour and incubated with the appropriate concentration of primary antibodies (1:1000) at 4°C overnight. The membranes were cut in pieces according to the band size before incubation with primary antibodies. The membranes were subsequently incubated with an appropriate concentration of secondary antibodies (1:15,000) for 1 hour at room temperature in the dark. The protein bands in the membrane were visualized by a UVP Bio Imaging System. GAPDH proteins were evaluated by the same procedure. Stripping Buffer （CWBIO,Stripping Buffer,CW0056M）was used for the recycle of deproteinized membrane in our experiment. Steps are as follows: Wash membrane with TBST for three times, 10 minutes each. Immerse it into appropriate volume of WB Stripping Buffer, incubate at room temperature for 30 minutes and shake slowly. Take out membrane with tweezer. Elute with TBST once and wash for 5 minutes. Seal with 5% defatted milk powder and incubate with the appropriate concentration of next primary antibodies (1:1000) at 4°C overnight. Finally, the reported data for Bax (Proteintech^®^Bax rabbit polyclonal antibody, 50599‐2‐lg), Bcl2 (Proteintech^®^ Bcl2 rabbit polyclonal antibody, 12789‐1‐AP), caspase‐3 (Proteintech^®^ caspase‐3 rabbit polyclonal antibody, 19677‐1‐AP), cleaved caspase‐3 (Cell Signaling Technology^®^Cleaved Caspase‐3 rabbit mAb, 9664), p65NF‐κB (Proteintech^®^ P65 rabbit polyclonal antibody, 10745‐1‐AP), p‐p65NF‐κB p38 (Proteintech^®^p38 rabbit polyclonal antibody, 14064‐1‐AP), p‐p38 (Cell Signaling Technology^®^Phospho‐p38 MAPK XP^®^ rabbit mAb, 4511), ERK1/2 (Cell Signaling Technology^®^p44/42 MAPK rabbit mAb, 4695), p‐ERK1/2 (Phospho‐p44/42 MAPK (Erk1/2)XP^®^rabbit mAb, 4370), JNK (Proteintech^®^JNK rabbit polyclonal antibody, 24164‐1‐AP) and p‐JNK (Cell Signaling Technology^®^Phospho‐SAPK/JNK rabbit mAb, 4668) band densities were normalized to GAPDH (Cell Signaling Technology^®^ GAPDH rabbit mAb, 2118). The data were analysed by ImageJ software.

### 2.10 Data analysis and statistics

All data are expressed as the means ± SD. Statistical significance was assessed by Student's *t* test for two groups and one‐way ANOVA for comparisons of more than two groups. Differences where *P* < 0.05 were defined as statistically significant.

## 3 RESULTS

### 3.1 CGA has cardioprotective effects and mitigates the overexpression of TNF‐α in a TAC heart failure mouse model

**Figure 1:**
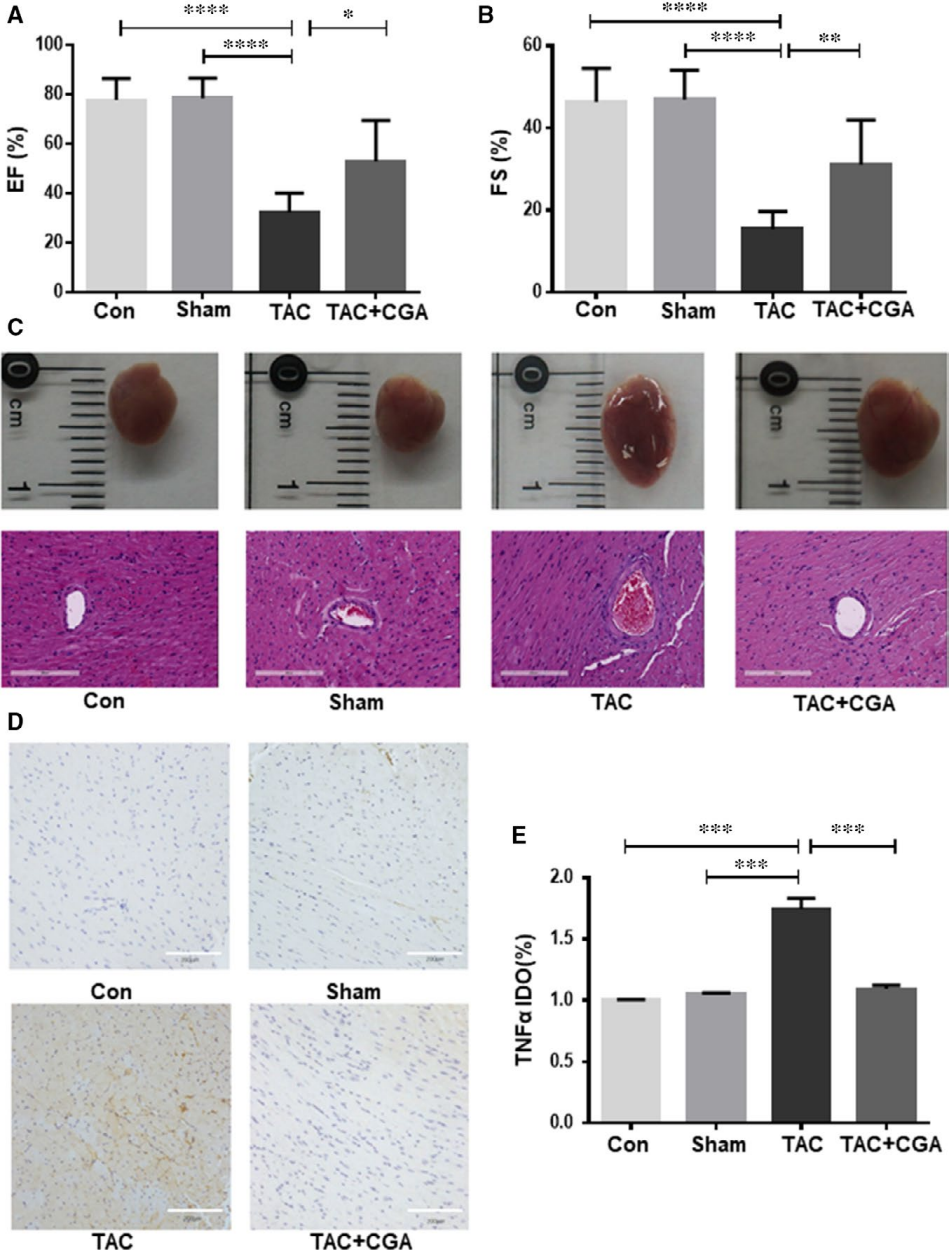
Chlorogenic acid (CGA) has cardioprotective effects and mitigates the overexpression of TNF‐α in transverse aortic constriction (TAC), a heart failure mouse model. Representative echocardiograph evaluation of the ejection fraction (EF) and left ventricular fraction shortening (FS) from various treatment groups, including control group (n = 6); sham group (n = 6); TAC group (n = 6); and TAC + CGA group(n = 9) (A,B). Spherical shape of heart from different treatment groups and haematoxylin and eosin staining (HE) (Scale bar = 200 μm) (C). TNF‐α expression levels in mouse hearts from different treatment groups detected by immunohistochemical staining (Scale bar = 200 μm) (D). Quantitative analysis of TNF‐α IOD rate (E). Results are expressed as the means ± SD from three independent experiments. *P < 0.05, **P < 0.01, ***P < 0.001, ****P < 0.0001 significantly different between two groups

We used echocardiography to evaluate the left ventricular function in blank control, sham‐operated, TAC‐induced heart failure model and CGA‐treated groups, and showed that the EF and FS in the TAC‐induced heart failure model group was significantly reduced compared with the control and sham groups. After treatment with CGA, EF and FS were significantly improved, indicating that CGA improved the left ventricular function in TAC‐induced heart failure (Figure 1A,B, Table 1).

**Table 1:** Ejection fraction and fractional shortening for different groups

Group	N	EF (%)	FS (%)
Con	6	77.78 ± 8.71	46.31 ± 8.13
Sham	6	78.58 ± 7.93	46.86 ± 7.16
TAC	6	32.36 ± 7.73	15.40 ± 4.24
TAC + CGA	9	52.90 ± 16.63	31.05 ± 10.90

Abbreviations: CGA, chlorogenic acid; TAC, transverse aortic constriction.

Haematoxylin and eosin staining demonstrated that the myocardium of control and sham groups was normal and that cardiomyocytes were present in an orderly arrangement. However, the TAC model group showed cardiomyocyte hypertrophy, derangement and massive necrosis, as well as muscle fibre dissolution and normal structure loss. In the CGA treatment groups, the cardiomyocytes were arranged neatly and maintained their original morphology. The ventricular cavity of the TAC model group was larger than in the control and sham groups, and CGA treatment significantly recovered enlargement of the ventricular cavity (Figure 1C). These results suggest that CGA had a protective effect against TAC‐induced cardiovascular structure and function impairment.

To further explore the effect of CGA on TAC‐induce heart failure, the expression of TNF‐α, a biomarker of heart failure, was assessed by immunohistochemical staining. This confirmed that CGA significantly suppressed the TAC‐induced upregulation of TNF‐α, suggesting CGA has cardioprotective effects and might mitigate the overexpression of TNF‐α in a TAC heart failure mouse model (Figure 1D,E).

### 3.2 TNF‐α promotes cell apoptosis in hiPSC‐CMs

**Figure 2:**
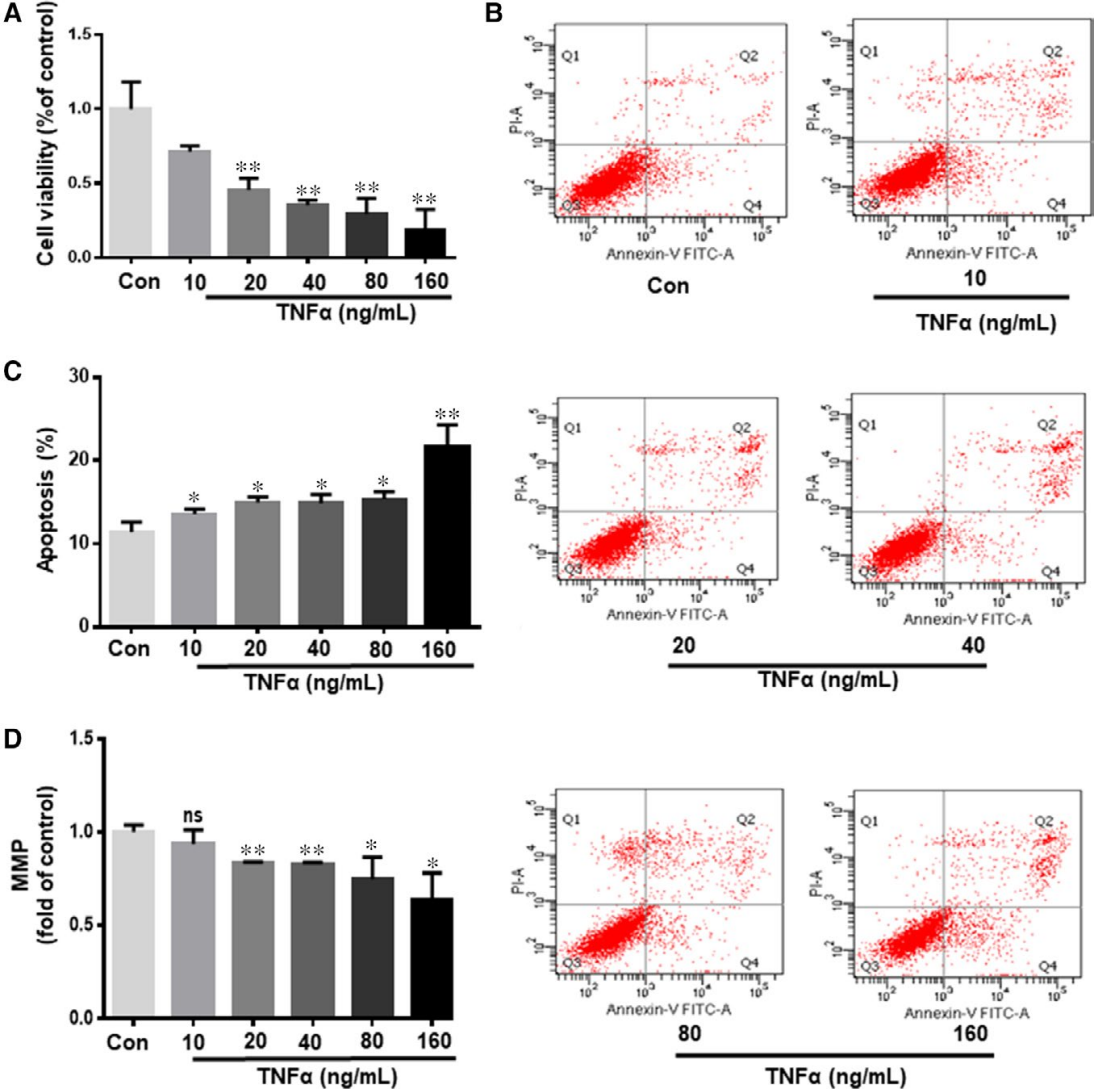
TNF‐α promotes cell apoptosis in human‐induced pluripotent stem cell derived cardiomyocytes. hiPSC‐CMs were treated with various doses of TNF‐α (10, 20, 40, 80 or 160 ng/mL) for 24 h. Then the viability of hiPSC‐CMs was evaluated by CCK‐8 assay (A). TNF‐α–induced hiPSC‐CM apoptosis was detected by Annexin V/PI assay double staining assay (B). Quantitative analysis of apoptosis rate (C). MMP was measured by JC‐1 (D). Values are expressed as the means ± SD from three independent experiments. *P < 0.05, **P < 0.01, ***P < 0.001, ****P < 0.0001 significantly different between two groups

We used hiPSC‐CMs to explore whether CGA might have cardioprotective effects against TNF‐α–induced apoptosis of myocardial cells. hiPSC‐CMs were pre‐conditioned with various concentrations of TNF‐α (10‐160 ng/mL) at 24 hours and their viability was evaluated by CCK‐8 assay. The results indicated that TNF‐α treatment led to a dose‐dependent reduction in cell viability. Specifically, the viability of hiPSC‐CMs was significantly decreased to approximately 40% after 24 hours and TNF‐α treatment at concentrations up to 40 ng/mL (Figure 2A). To identify whether TNF‐α–induced cell death was apoptosis, Annexin V/PI double staining assay was performed with hiPSC‐CMs treated with different concentrations of TNF‐α for 24 hours. The number of apoptotic hiPSC‐CMs was obviously increased after TNF‐α treatment and was dose dependent (Figure 2B,C).

TNF‐α–induced hiPSC‐CM injury might result in opening of the mitochondrial permeability transition pore (mPTP) of the inner mitochondrial membrane, leading to mitochondrial membrane depolarization as well as pro‐apoptotic substance release.^35^ We performed JC‐1 staining to evaluate the mitochondrial membrane potential and TNF‐α–induced mitochondrial injury. The MMP of hiPSC‐CMs subjected to TNF‐α was markedly decreased and this was dose dependent (Figure 2D). According to the above experiment, we used 40 ng/mL TNF‐α for the following experiments.

### 3.3 CGA increases cell viability and attenuates TNF‐α–induced apoptosis in hiPSC‐CMs

**Figure 3:**
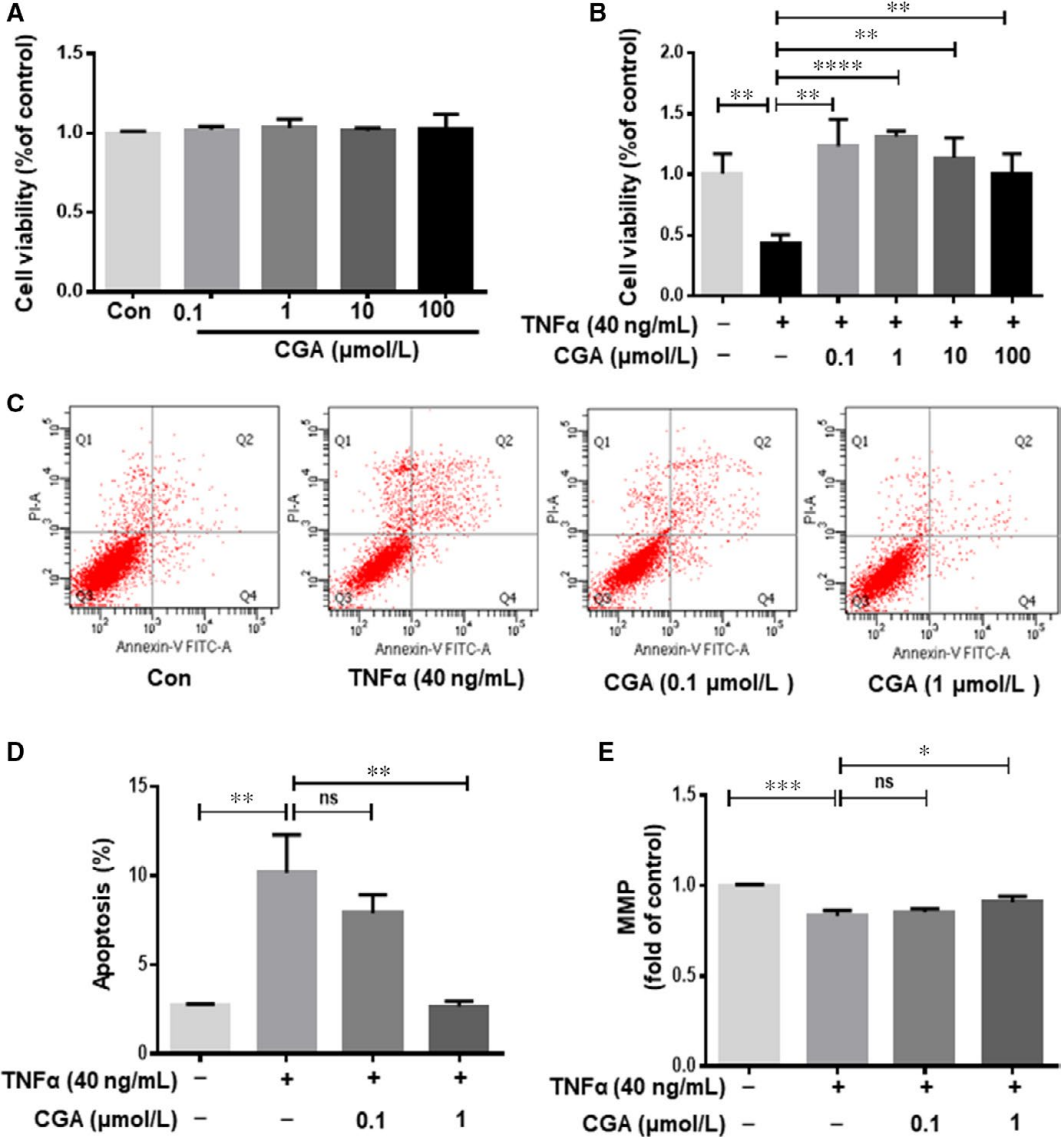
Chlorogenic acid increases cell viability and attenuates TNF‐α–induced apoptosis in human‐induced pluripotent stem cell derived cardiomyocytes. hiPSC‐CMs were treated with various doses of CGA (0.1, 1, 10, and 100 μmol/L) for 12 h, then the viability of hiPSC‐CMs was evaluated by CCK‐8 assay (A). hiPSC‐CMs were incubated with CGA (0.1 μmol/L or 1 μmol/L) for 12 h before TNF‐α treatment for 24 h. Then the cell viability of hiPSC‐CMs was assessed by CCK‐8 assay (B). CGA inhibited TNF‐α–induced hiPSC‐CMs apoptosis detected by Annexin V/PI assay double staining assay (C). Quantitative analysis of apoptosis rate (D). MMP was measured by JC‐1 (E). Results are expressed as the means ± SD from three independent experiments. *P < 0.05, **P < 0.01, ***P < 0.001, ****P < 0.0001 significantly different between two groups

To explore the effect of CGA on TNF‐α–induced hiPSC‐CMs apoptosis, we assessed whether CGA has cytotoxic effects on hiPSC‐CMs. No changes in cell viability were observed for hiPSC‐CMs after CGA treatment for 12 hours, even at the maximal dose of CGA (Figure 3A). Moreover, CGA effectively ameliorated the decline in hiPSC‐CM viability induced by TNF‐α (Figure 3B). To further investigate whether CGA attenuated TNF‐α–induced apoptosis in hiPSC‐CMs, Annexin V/PI double staining was employed to quantify apoptosis in cells from different groups. The percentage of apoptosis was significantly higher in the TNF‐α treatment group compared with the control group. However, this phenomenon was significantly attenuated by pre‐treatment with CGA (Figure 3C,D). These results indicate that CGA protects hiPSC‐CMs from TNF‐α–induced apoptosis. Moreover, the MMP of hiPSC‐CMs subjected to TNF‐α was markedly decreased and this trend was significantly reversed by pre‐treatment with CGA (Figure 3E).

**Figure 4:**
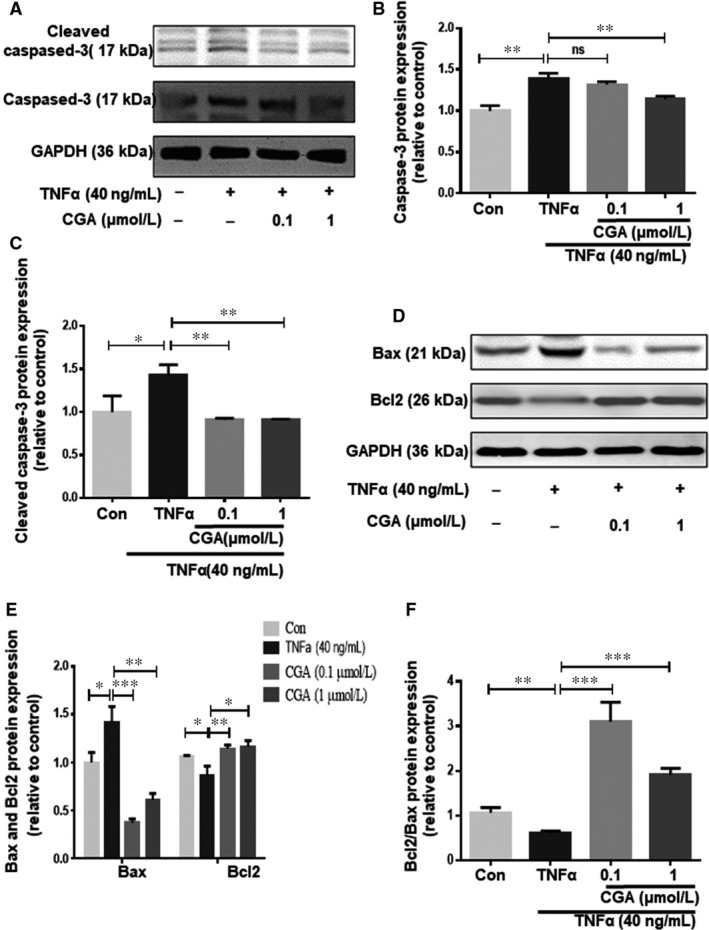
The protective effect of CGA on TNF‐α–induced hiPSC‐CM apoptosis. hiPSC‐CMs were incubated with CGA (0.1 μmol/L or 1 μmol/L) for 12 h before TNF‐α treatment for 24 h. The protein levels of caspase‐3 and cleaved caspase‐3 were detected by western blot (A). Densitometric analysis was conducted to quantify caspase‐3 and cleaved caspase‐3 protein levels (B,C). The levels of Bax and Bcl2 were assessed by western blot (D). Quantitative analysis of Bax, Bcl2 and Bcl2/Bax expression (E,F). Data are the means ± SD from three independent experiments. *P < 0.05, **P < 0.01, ***P < 0.001, ****P < 0.0001 significantly different between two groups

Caspase‐3, activated through the proteolytic processing of procaspase‐3 into 12 and 17 kDa subunits, which are early markers of apoptosis, in turn activates the mitochondrial apoptotic pathway. In addition, the apoptotic process includes a variety of regulatory genes, of which, the Bcl2 protein family serves as a crucial regulatory factor in the mitochondrial apoptotic pathway and is comprised of death inhibitors (Bcl2,bcl‐xL) and death activators (Bax, Bak). These regulate the release of (pro)apoptotic intermembrane proteins. To further characterize the anti‐apoptotic effect of CGA, the protein expressions of Bcl2, Bax, caspase‐3 and cleaved caspase‐3 were determined by western blot. CGA pre‐treatment significantly suppressed the TNF‐α–induced upregulation of caspase‐3 and cleaved caspase‐3 protein expressions (Figure 4A‐C). Furthermore, the decrease in the Bcl2/Bax protein expression ratio induced by TNF‐α was inhibited by CGA pre‐treatment (Figure 4D‐F).

### 3.4 Role of NF‐κB in the protective effect of CGA against TNF‐α injured cardiomyocytes

**Figure 5:**
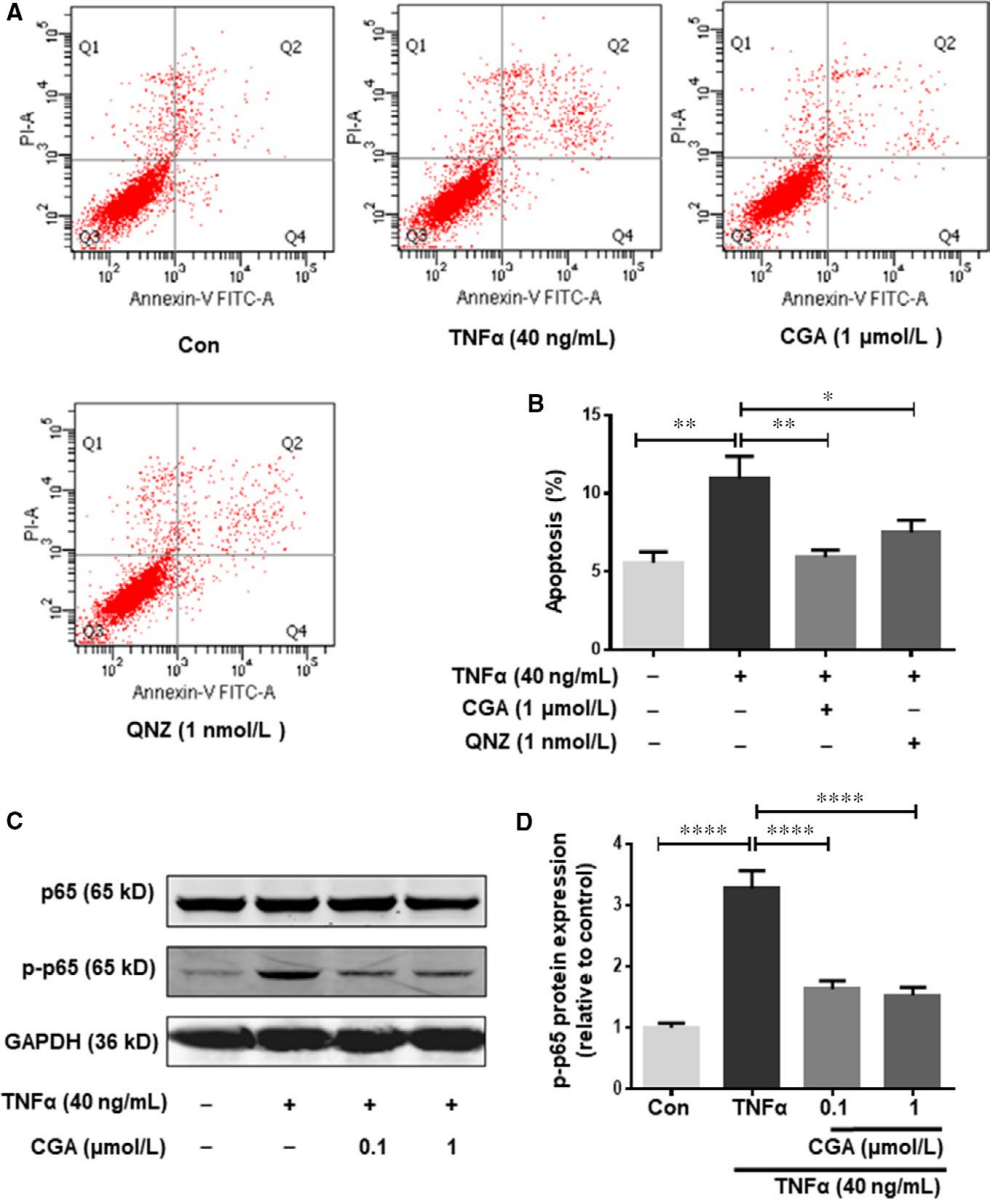
Role of NF‐κB in the protective effect of chlorogenic acid against TNF‐α injured cardiomyocytes. hiPSC‐CMs were treated with QNZ (1 nmol/L) for 4 h or CGA (0.1 μmol/L or 1 μmol/L) for 12 h prior to incubation with TNF‐α for 24 h. Then the apoptosis rate of hiPSC‐CMs was detected by Annexin V/PI staining, and the quantitative analysis of hiPSC‐CMs apoptosis rate is shown (A, B). Western blot analysis was conducted to examine phosphorylated NF‐κB p65 and NF‐κB p65 (C). Quantitative analysis of phosphorylated NF‐κB p65 and NF‐κB p65 expression (D). Data are the means ± SD from three independent experiments. *P < 0.05, **P < 0.01, ***P < 0.001, ****P < 0.0001 significantly different between two groups

NF‐κB plays a vital role in cell apoptosis when exposed to different stimuli, such as TNF‐α. NF‐κB transcription can ultimately result in cell apoptosis. To further investigate whether the NF‐κB signalling pathway regulates the anti‐apoptotic effects of CGA, we treated hiPSC‐CMs with an NF‐κB inhibitor (QNZ) for 4 hours before TNF‐α treatment. Annexin V/PI double staining assays showed that QNZ inhibited TNF‐α–induced hiPSC‐CMs apoptosis (Figure 5A,B). These results indicated that the NF‐κB signalling pathway was involved in TNF‐α–induced hiPSC‐CMs apoptosis. Furthermore, data from the Annexin V/PI double staining assay indicated that CGA inhibited TNF‐α–induced cardiomyocyte apoptosis to a greater degree than the NF‐κB inhibitor QNZ. To confirm and analyse the effects of the NF‐κB signalling pathway in the CGA inhibition of TNF‐α–induced apoptosis, the protein expressions of p‐NF‐κB/p65 and NF‐κB/p65 were examined by western blot. The expression levels of NF‐κB/p65 remained relatively unaltered in TNF‐α treated and CGA‐pre–treated groups, but the protein expression level of p‐NF‐κB/p65 was higher in TNF‐α‐treated hiPSC‐CMs than in untreated hiPSC‐CMs (control) (Figure 5C,D). However, p‐NF‐κB/p65 was triggered by TNF‐α, and inhibited by CGA. These results suggested that the anti‐apoptotic effects of CGA were related to its ability to inhibit p‐NF‐κB/p65.

### 3.5 CGA attenuates TNF‐α–induced cardiomyocyte apoptosis by inhibiting JNK

**Figure 6:**
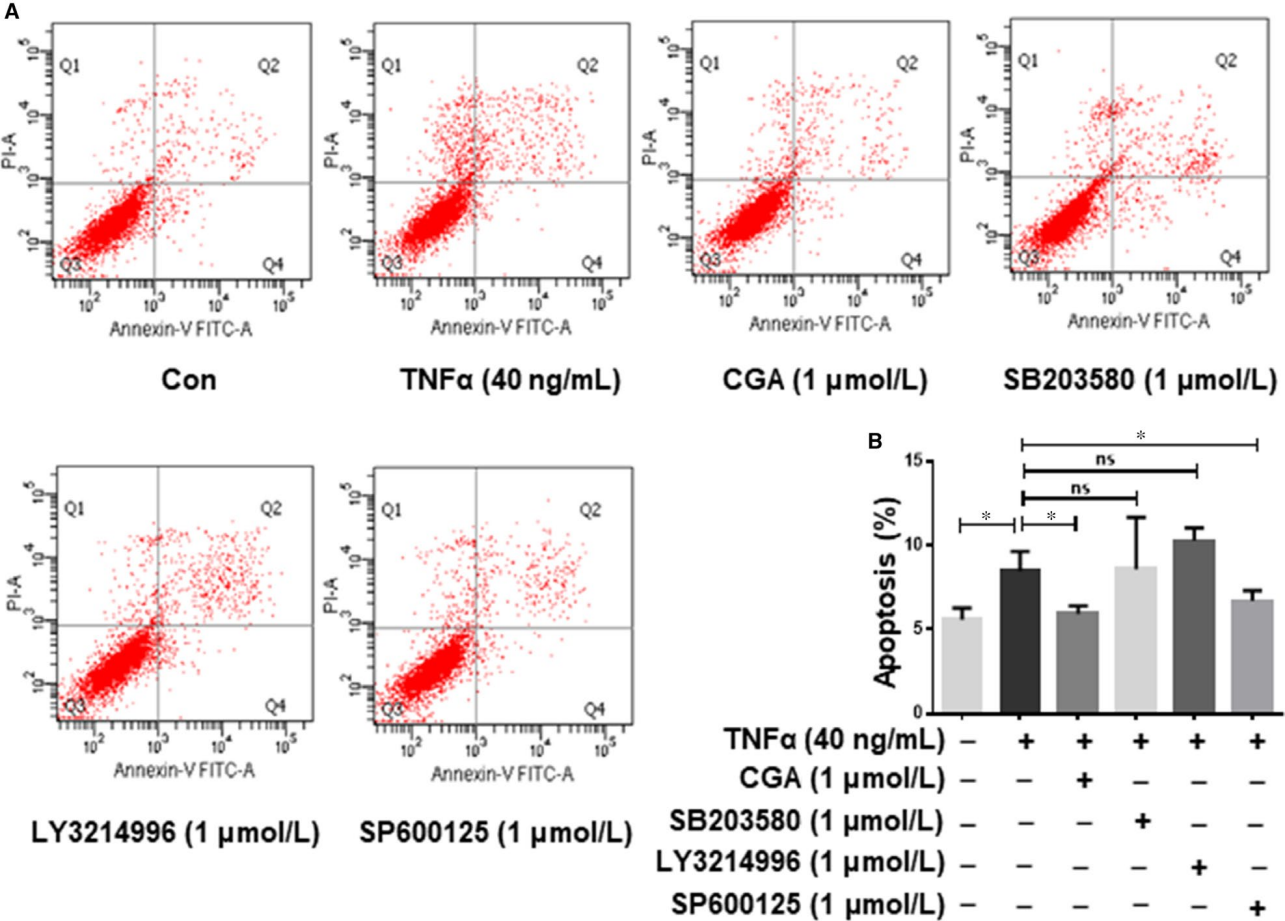
Chlorogenic acid attenuation of TNF‐α–induced cardiomyocyte apoptosis is independent of ERK1/2. hiPSC‐CMs were treated with SB203580 (1 μmol/L), LY3214996 (1 μmol/L) or SP600125 (1 μmol/L) for 4 h, or CGA (1 μmol/L) for 12 h prior to incubation with TNF‐α for 24 h. Then the apoptosis rate of hiPSC‐CMs was detected by Annexin V/PI staining, and the quantitative analysis of hiPSC‐CMs apoptosis rate is shown (A,B). Data are the means ± SD from three independent experiments. *P < 0.05, **P < 0.01, ***P < 0.001, ****P < 0.0001 significantly different between two groups

**Figure 7:**
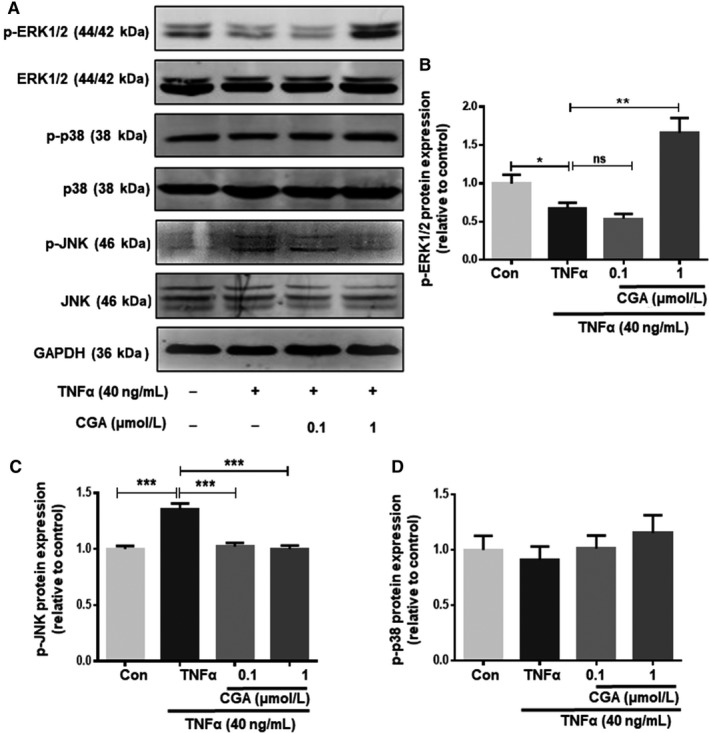
The protective effects of CGA on TNF‐α–induced apoptosis in hiPSC‐CMs are mediated by inhibiting JNK. hiPSC‐CMs were treated with CGA (0.1 μmol/L or 1 μmol/L) for 12 h prior to incubation with TNF‐α for 24 h. The levels of phosphorylated JNK, JNK, phosphorylated p38, p38, phosphorylated ERK and ERK were determined by western blot (A). Quantitative analysis of phosphorylated ERK and ERK expression (B). Quantitative analysis of phosphorylated JNK and JNK(C). Quantitative analysis of phosphorylated p38 and p38 expression (D). Results are the means ± SD from three independent experiments. *P < 0.05, **P < 0.01, ***P < 0.001, ****P < 0.0001 significantly different between two groups

MAPKs, including p38MAPK, ERK1/2 and JNK, are related to the regulation of many cellular events, such as cell proliferation, survival and apoptosis, which are specifically activated by TNF‐α. To explore the molecular mechanism by which the MAPK pathway is involved in the effect of CGA on TNF‐α–induced apoptosis, we separately treated hiPSC‐CMs with a p38MAPK (SB203580) inhibitor, ERK1/2 inhibitor (LY3214996) and JNK inhibitor (SP600125) for 4 hours before TNF‐α treatment. Annexin V/PI double staining assays revealed that TNF‐α–induced apoptosis was reversed by the JNK inhibitor (SP600125) but not by the ERK1/2 inhibitor (LY3214996) or p38MAPK (SB203580) inhibitor (Figure 6A,B). These results indicate that JNK is involved in TNF‐α–induced hiPSC‐CMs apoptosis. Then, we further examined the activation of p38MAPK, ERK1/2 and JNK by western blot. TNF‐α treatment did not activate ERK1/2, and CGA increased the phosphorylation of ERK1/2 suggesting that ERK1/2 is irrelevant for TNF‐α–induced injury in hiPSC‐CMs and the protective effects of CGA (Figure 7A,B). The phosphorylation level of JNK was significantly enhanced in TNF‐α treated hiPSC‐CMs compared with control hiPSC‐CMs (Figure 7A,C), and pre‐treatment with CGA prevented the activation of p‐JNK. The expression levels of p‐p38MAPK and p38MAPK were unchanged in TNF‐α–treated hiPSC‐CMs with or without CGA (Figure 7A,D). These results demonstrate that the JNK pathway has a crucial role in facilitating the anti‐apoptotic effects of CGA.

## 4 DISCUSSION

The vital role of TNF‐α in the pathogenesis of heart failure has been widely accepted, and TNF‐α might also provide useful prognostic information as a biomarker for heart failure.^36^, ^37^ It should be noted that low levels of TNF‐α in heart tissues might to be essential to protect the myocardium from injury, whilst higher levels of systemic TNF‐α result in the development of ventricular dysfunction.^38^ Experimental studies with mammalian cardiomyocytes indicated that cardiac‐specific overexpression of TNF‐α recapitulated the phenotype of heart failure.^39^ Given the important function of TNF in the pathogenesis of heart failure, it was speculated that suppression of the overexpression of TNF‐α might have a therapeutic effect in patients suffering from heart failure.^12^ CGA, the main effector component of *Lonicerae Japonicae Flos*, has numerous pharmacological effects, including anti‐inflammatory and anti‐apoptotic effects. In the current study, evidence from our in vivo study demonstrated that CGA has cardioprotective effects and mitigated TNF‐α–induced toxicity in a TAC heart failure mouse model. In the present in vitro study, we further showed that CGA attenuated TNF‐α–induced apoptosis in hiPSC‐CMs via multiple mechanisms. CGA inhibited TNF‐α–induced hiPSC‐CMs apoptosis by controlling the mitochondrial apoptotic pathway. In addition, CGA specifically regulated the activation of NF‐κB/p65 and JNK signals to exert anti‐apoptotic effects.

High levels of inflammasome TNF‐α might disrupt mitochondrial membrane potential and provoke opening of the mPTP in pathological conditions.^40^ Durable mPTP opening as well as membrane potential depolarization can result in the release of mitochondrial death factor, which induces the activation of caspase‐3 and cell apoptosis. Caspase‐3 is a crucial protein involved in classic apoptosis, which is activated by the mitochondrial apoptotic pathway.^41^, ^42^ Its activation forms cleaved caspase‐3, a pro‐apoptotic marker, and initiates apoptosis.^43^ Furthermore, the mitochondrial apoptotic pathway is regulated via the ratio of pro‐apoptotic to anti‐apoptotic proteins of the Bcl2 family.^44^ Bcl2, which resides in the mitochondrial membrane, was first discovered to have anti‐apoptotic functions. It forms heterodimers with pro‐apoptotic protein Bax and blocks the mitochondrial apoptosis pathway.^45^ Our data indicated that TNF‐α induced the mitochondrial apoptosis pathway by upregulating the levels of caspase‐3 and cleaved caspase‐3 and destroying the balance between Bcl2 and Bax. However, pre‐treatment with CGA strongly attenuated this condition. The data in our study clearly revealed that CGA inhibited TNF‐α–induced hiPSC‐CMs apoptosis by controlling the mitochondrial apoptotic pathway.

NF‐κB is a transcription factor that regulates a variety of genes that participate in several crucial physiological processes such as survival, inflammation or immune responses.^46^ Recent studies demonstrated that NF‐κB has cardioprotective effects though repression of apoptotic cell death induced by hypoxia or myocardial injury.^47^, ^48^, ^49^ In particular, the sustained activation of NF‐κB accelerates heart failure by eliciting signals that induce chronic inflammation through increased levels of TNF‐α, resulting in endoplasmic reticulum stress responses or cell death.^50^ Signalling pathway that activate the NF‐κB family are characterized as canonical and non‐canonical pathway. The canonical signalling pathway promotes p65/p50 NF‐κB dimers, and the non‐canonical signalling promotes RelB/p52 dimers.^51^ TNF‐α activates the canonical NF‐κB signalling pathway.^50^ A previous study indicated that p50^−/−^mice were resistant to TNF‐α–induced cardiomyopathy and had enhanced cardiac function after myocardial infarction.^52^, ^53^ Therefore, targeting NF‐κB responses are essential for the development novel therapeutic interventions for heart failure following myocardial injury. A previous study found that the NF‐κB inhibitor BAY11‐7082 strongly decreased infarct size and enhanced myocardial function by reducing inflammation and apoptosis.^54^ Moreover, Li et al found that pre‐treatment with CGA could inhibit iso‐induced cardiac hypertrophy through blocking NF‐κB signalling pathway. Their results mainly indicated that CGA pre‐treatment could inhibited the translocation of NF‐κB into the nucleus.^55^ The results of our study further elucidated the effects of CGA on NF‐κB, whereby phosphorylated NF‐κB/p65 was activated after TNF‐α stimulation of hiPSC‐CM. CGA pre‐treatment remarkably reversed this effect. These observations demonstrated that CGA specifically attenuated the TNF‐α–induced activation of NF‐κB/p65 in hiPSC‐CMs to exert an anti‐apoptotic effect.

MAPKs are protein serine/threonine kinases comprising three major signalling pathways, namely ERK1/2, JNK/SAPK and p38MAPK, which participate in cell proliferation, differentiation and apoptosis.^56^ The effect of p38 MAPK and JNK/SAPK on apoptosis remains controversial because both pro‐ and anti‐apoptotic influences have been observed dependent upon cell type and apoptotic stimuli. However, the activation of p38 and JNK/SAPK is thought to promote TNF‐α–induced cardiomyocyte apoptosis.^57^ Conversely, the ERK1/2 signal pathway participates in regulating cardiac myocyte growth and provides cell protection.^19^ Our results show that p‐JNK was activated by TNF‐α, and that the activation of p‐JNK was blocked by pre‐incubation with CGA, suggesting CGA protects hiPSC‐CM from TNF‐α–induced apoptosis by inhibiting the JNK signalling pathway. Our findings are consistent with previous research in which JNK was shown to be important in ischemic diseases mostly by regulating apoptosis.^58^

**Figure 8:**
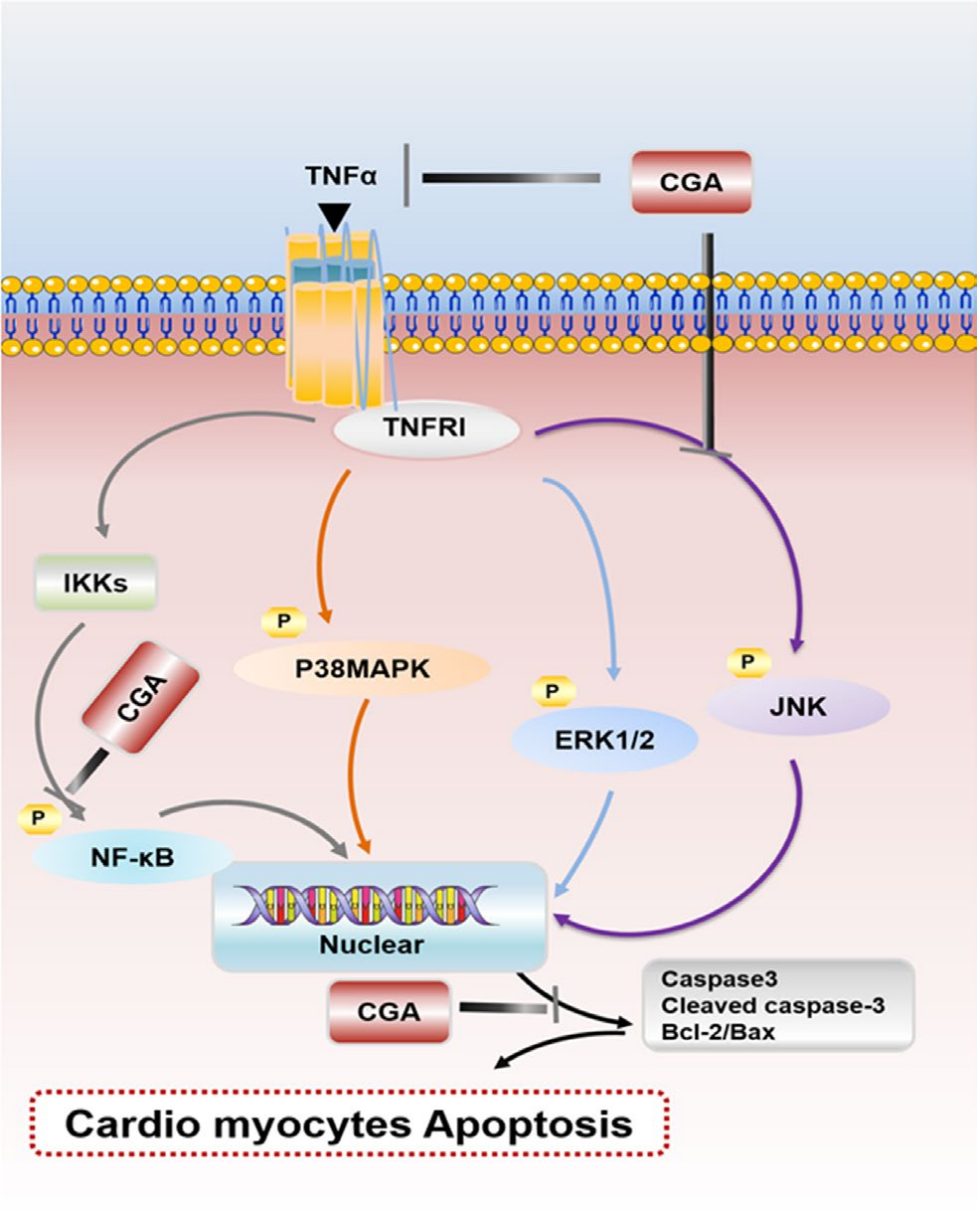
Schematic illustration of the protective mechanisms of CGA against TNF‐α–induced cardiomyocyte apoptosis

In conclusion, this study provides initial evidence that CGA effectively regulates the mitochondrial apoptotic pathway and markedly inhibits the activation of NF‐κB and JNK. These effects might have vital roles in the mechanism of CGA‐mediated myocardial protection (Figure 8). If these effects of CGA are validated in clinical trials, it might be a promising agent for preventing and treating heart failure.

## CONFLICT OF INTEREST

The authors have no conflict of interest to declare.

## AUTHOR CONTRIBUTIONS

Lei Tian, Cong‐Ping Su, Qing Wang, Fu‐Jian Wu and Rui Bai contributed to the study design, data collection, study execution and preparation of the manuscript; Shu‐Zhen Guo, Feng Lan, Wen‐Jing Lu and Wei Wang were responsible for conception, design, and financial support; and Hui‐Min Zhang and Jin‐Ying Liu contributed to data analysis. All authors read and approved the final manuscript.

## DATA AVAILABILITY STATEMENT

The data that support the findings of this study are available from the corresponding author upon reasonable request.
