# Supplementary figures and images for: Chlorogenic acid: A potent molecule that protects cardiomyocytes from TNF‐α–induced injury via inhibiting NF‐κB and JNK signals
Source: J Cell Mol Med. 2019 Apr 29;23(7):4666–78. doi: 10.1111/jcmm.14351 (PMC6584503; doi:10.1111/jcmm.14351)

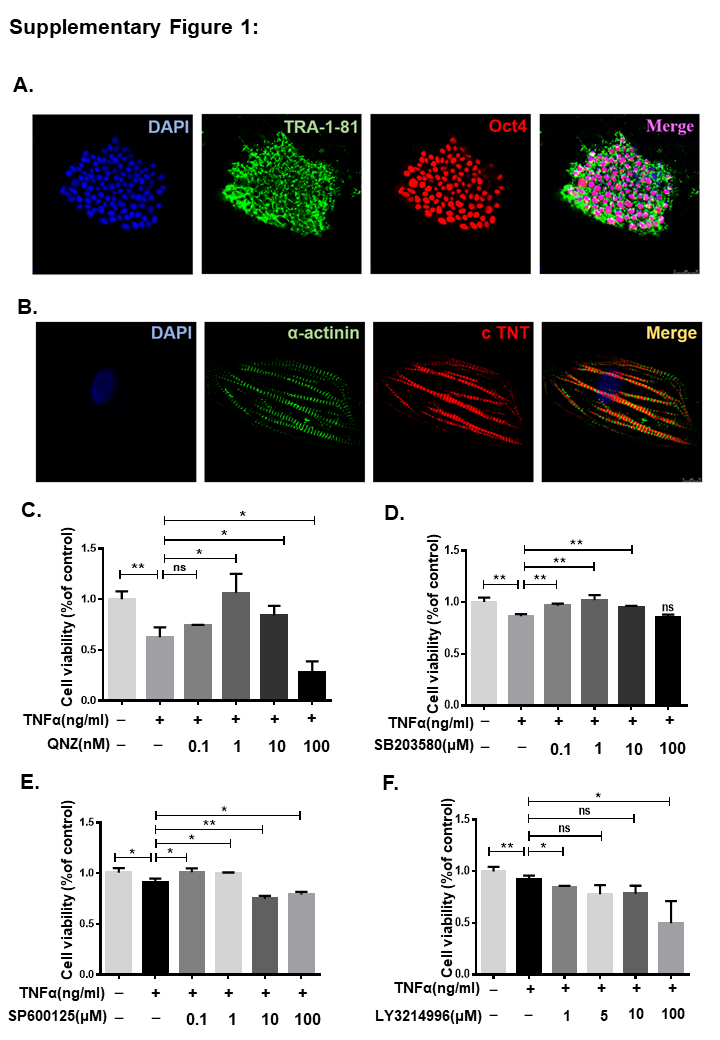

Supplement: Supplementary file 1 [file JCMM-23-4666-s001.TIF]
